# BNT162b2 mRNA SARS-CoV-2 Vaccine Elicits High Avidity and Neutralizing Antibodies in Healthcare Workers

**DOI:** 10.3390/vaccines9060672

**Published:** 2021-06-18

**Authors:** Federico Pratesi, Teresita Caruso, Davide Testa, Tiziano Tarpanelli, Alessandra Gentili, Davide Gioè, Paola Migliorini

**Affiliations:** 1 Department of Clinical and Experimental Medicine, University of Pisa, 56126 Pisa, Italy; teresita.caruso@outlook.it (T.C.); testa93davide@gmail.com (D.T.); paola.migliorini@med.unipi.it (P.M.); 2 Dia.Metra Srl—Immuno Diagnostic System, Spello, 06038 Perugia, Italy; tiziano.tarpanelli@idsplc.com (T.T.); alessandra.gentili@idsplc.com (A.G.); davide.gioe@idsplc.com (D.G.)

**Keywords:** SARS-CoV-2, vaccine, immune response, neutralizing antibodies, antibody avidity

## Abstract

The BNT162b2 vaccine, containing lipid nanoparticles-formulated mRNA encoding the full-length spike protein of SARS-CoV-2, has been employed to immunize health care workers in Italy, administered in two doses 21 days apart. In this study, we characterized the antibody response induced by the BNT162b2 vaccine in a group of health care workers, tested at baseline, after the first dose and after the booster. Thirty-nine subjects without previous exposure to SARS-CoV-2 were vaccinated with the BNT162b2 vaccine. IgM, IgG, and IgA anti-receptor binding domain (RBD) were tested by ELISA. Neutralizing antibodies were evaluated testing the inhibition of RBD binding to ACE2. Antibody avidity was measured by urea avidity ELISA. IgM anti-RBD are produced after the first dose of vaccine and persist after the booster. IgG and IgA anti-RBD antibodies are detected in high amounts in all the subjects after the first dose and further increase after the booster. A few subjects, already after the first dose, produce antibodies inhibiting RBD interaction with ACE2. After the booster, high levels of inhibitory antibodies are detected in all the subjects. Affinity maturation takes place with boosting and IgG anti-RBD avidity increases with the number of immunizations. A less pronounced increase is observed with IgA. These data indicate that the BNT162b2 vaccine can induce high levels of protective antibodies of high avidity in vaccinated subjects; both IgG and IgA anti-RBD antibodies are produced. Further studies are needed to evaluate antibody persistence over time.

## 1. Introduction

The COVID-19 pandemic outbreak, caused by the novel severe acute respiratory syndrome Coronavirus 2 (SARS-CoV-2), emerged at the end of 2019 in Wuhan, China and rapidly spread all around the world, still representing a threat for the public health.

Social distancing, partial or total lockdowns, and public education about personal protection have been exploited by all countries and helped to limit the virus’ transmission and to relieve the burden on hospital wards, but were far from representing a solution for the challenge launched by SARS-CoV-2.

The best strategy to fight the pandemic is represented by a global vaccination campaign able to administer safe and effective vaccines for the protection of individuals, to achieve sufficient herd immunity to ultimately control the COVID-19 pandemic [1].

Facing such a challenge, SARS-CoV-2 vaccines have been developed at a very rapid pace thanks to the previous knowledge on the role of spike protein in SARS infection, the availability of the SARS-CoV-2 genetic structure and the evolution of nucleic acid vaccine technology platforms.

The extraordinary efforts made by every country during the COVID-19 pandemic, together with development activities run in parallel and not sequentially, allowed this goal to be achieved in a remarkably short time.

mRNA platform technology has allowed the rapid manufacturing of SARS-CoV-2 vaccines, and currently, two of them (BNT162b2 and mRNA-1273) have been validated for emergency use all over the world [2,3,4]. Both contain lipid nanoparticles-formulated mRNA encoding the full-length spike protein of SARS-CoV-2. BNT162b2 (also known as Comirnaty or tozinameran) has been employed to immunize health care workers in Italy, administered in two doses 21 days apart.

Successful vaccination regimens result in antibody responses that are robust in both quantity and quality. So far, a number of studies evaluated the anti-Spike or anti-receptor binding domain (RBD) antibody titer [5,6,7] and their neutralizing activity [8], but little is known about the avidity of antibodies elicited by the mRNA SARS-CoV-2 vaccine [9]. Moreover, the extent of affinity maturation in COVID-19 patients and its impact on the clinical course of the disease is still a subject of active investigation [10,11,12].

In this study, we characterized the antibody response induced by the BNT162b2 vaccine in a group of health care workers, tested at baseline, after the first dose and after the booster, obtaining evidence for a high efficacy of the vaccine in inducing neutralizing high avidity antibodies.

## 2. Materials and Methods

### 2.1. Subjects

This study has been conducted in accordance with the Helsinki Declaration as revised in 2013. Sera from voluntary health care workers undergoing BNT126b2 vaccination were obtained following a clinical protocol approved by the Ethical Committee of the Pisa University Hospital (Approval N° 17522). Whole blood was collected upon patient’s written informed consent a few days before the vaccine (T0), 10–12 days after the first dose (T1) and 21 days after the second (T2), and was fractionated; and sera were collected and kept frozen at −60 °C until use. Thirty-nine subjects without previous exposure to SARS-CoV-2 were recruited (27 females, 12 males; age 27–65 years; mean age 46.8 years). Two additional subjects previously exposed to SARS-CoV-2 but asymptomatic were also vaccinated and included in the study. Sera obtained from 17 patients recovered in intensive care units with positive SARS-CoV-2 PCR nasopharyngeal swab and clinical symptoms of COVID-19 were used as the control for IgG avidity assay.

### 2.2. Analysis of Anti-RBD Antibody Titers

For antibodies detection, polystyrene plates were coated overnight with recombinant Receptor Binding Domain (aa _319–541_) (RBD—a kind gift from Prof. Gianpietro Corradin, Universitè de Lausanne, Lausanne, Switzerland) at 2 ug/mL in PBS pH 7.4. After blocking with PBS pH 7.4, BSA 3%, sera diluted 1/100 in PBS pH 7.4, BSA 1%, and Tween-20 0.05% were incubated in duplicate on the plate for 2 h. After 3 washings with PBS Tween-20 0.05%, goat anti-human IgG HRP (A0293—Sigma Aldrich Merck KGaA, Darmstadt, Germany) diluted 1:5000 in PBS BSA 1% Tween-20 0.05% was added to the plates and incubated for 2 h. For IgM and IgA determination, goat anti-human IgM HRP (A0420—Sigma Aldrich) or goat anti-human IgA HRP (A0295—Sigma Aldrich) diluted 1:20,000 in PBS, BSA 1%, and Tween 0.05% were employed. After 3 washings with PBS Tween-20 0.05%, enzymatic activity was measured at 450 nm after TMB addition (T4444—Sigma Aldrich) and blocking by H_2_SO_4_ 1M.

Antibody titer was also evaluated by means of end point dilution, testing the binding to RBD of 21 representative subjects at T0, T1 and T2 time points, for IgG, IgM and IgA, starting from 1/100 and going on with 2-fold titration up to 1/12,800.

For each sample, we calculated the EPD as the highest dilution, giving an optical density of 3 standard deviations above the negative control, and we plotted on a graph the percentage of positive sera vs. reciprocal of EPD.

### 2.3. Analysis of Neutralizing Antibodies

To detect antibodies inhibiting the interaction between RBD and human Angiotensin Converting Enzyme 2 (ACE2) receptor, the SPIA (Spike Protein Inhibition Assay, Dia.Metra, Spello, Perugia, Italy) kit was employed according to manufacturer’s instructions. Briefly, the principle of the assay is a competitive/inhibition ELISA in which anti-SARS-CoV-2 neutralizing antibodies present in calibrator/controls and patient’s samples compete with a horseradish-peroxidase-conjugated ACE2 receptor for binding to SARS-CoV-2 wild type RBD coated on the microwell plate. Sera were tested in duplicate at 1:15 dilution and inhibition values were calculated using this formula:

% inhibition = [1−(Absorbance _Sample_)/(Absorbance _Calibrator_)] × 100 (1)

### 2.4. Evaluation of Antibody Avidity

**Figure 1:**
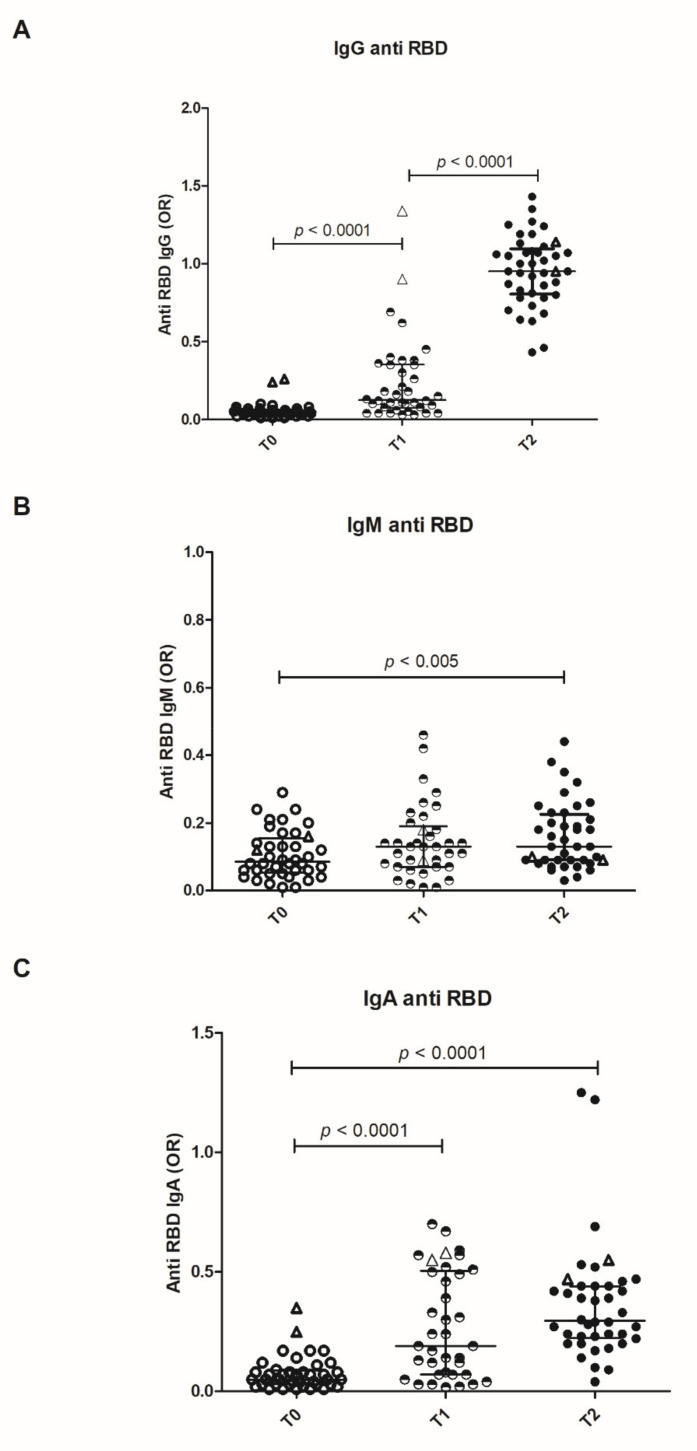
Distribution of anti-receptor binding domain immunoglobulins. Distribution of IgG (**A**), IgM (**B**) and IgA (**C**) anti-RBD antibodies at the baseline (T0), after the first dose (T1) and after the booster (T2) are shown. Results are expressed as odds ratio of an internal control (OR). Bars represent Median and interquartile range (IQR). The empty triangles represent the two vaccinated convalescent subjects. In these ones, IgG anti-RBD are detectable at the baseline and markedly increased after the first dose.

Antibody avidity was evaluated by means of an avidity ELISA, using urea as a chaotropic reagent. Briefly, after blocking and sera incubation, RBD plates were washed three times with PBS, Tween-20 0.05% and increasing concentrations of urea (urea 1M, 2M, 4M, 6M, and 8M) were added to the plate for 20 min at room temperature. After this step, washes with PBS, Tween 0.05%, anti-human IgG HRP-conjugated incubation, TMB addition and H_2_SO_4_ blocking were performed as described above for indirect anti-RBD IgG ELISA. Dilution curves were obtained for each sample and Avidity Index (AI) was calculated using the approach described by Polanec et al. [13]. The absorbance readings for each serum sample treated with different urea concentrations were converted to the percentage of untreated control OD remaining (% = (treated OD/untreated OD) × 100). The data were visualized with a graph, having on the *y*-axis, the percentage of the control OD for each sample and on the *x*-axis, the molar concentration of urea (Figure 1A—representative of a sample). The AI value determined using this approach corresponds with the extrapolated molar concentration of urea required to reduce the absorbance of the untreated control well by 50%.

### 2.5. Statistical Analysis

Data were analyzed and plotted using GraphPad Prism software (version 5.01; GraphPad Software Inc, San Diego, CA, USA). Antibody levels at different time points were compared in the three groups by the Kruskal–Wallis test. Correlations between different antibody distributions have been verified by Spearman’s rank correlation test. A *p* value < 0.05 was considered statistically significant.

## 3. Results

### 3.1. Analysis of Anti-RBD Immunoglobulins Titers in Vaccinated Healthcare Workers

IgG, IgA, and IgM antibodies were measured by a homemade solid phase assay on recombinant RBD. Antibody levels obtained by this test highly correlate with commercial anti-RBD assays (Spearman rank coefficient = 0.9349; *p* < 0.0001—Supplementary Figure S1).

IgM anti-RBDs are produced after the first dose of vaccine and persist after the booster. IgG and IgA antibodies are detected in high amounts in all the subjects after the first dose and further increase after the booster (Figure 1). A trend towards lower antibody levels with increasing age is observed. At variance with the unexposed individuals, two subjects that were previously infected showed high levels of anti-RBD IgG and IgA antibodies after the first dose, with no further increase after the booster (Figure 1). Antibody titers were also evaluated by means of endpoint dilution: IgG titers show a highly significative increase from T1 to T2, whereas IgM and IgA undergo a minor (albeit present) increase (Supplementary Figure S2).

### 3.2. Analysis of Neutralizing Antibodies in Vaccinated Healthcare Workers

To evaluate neutralizing antibodies, we tested the ability of sera to inhibit the interaction of SARS-CoV-2 RBD with the human host receptor angiotensin-converting enzyme 2 (ACE2), by means of the SPIA assay (Spike Protein Inhibition Assay, Dia.Metra).

**Figure 2:**
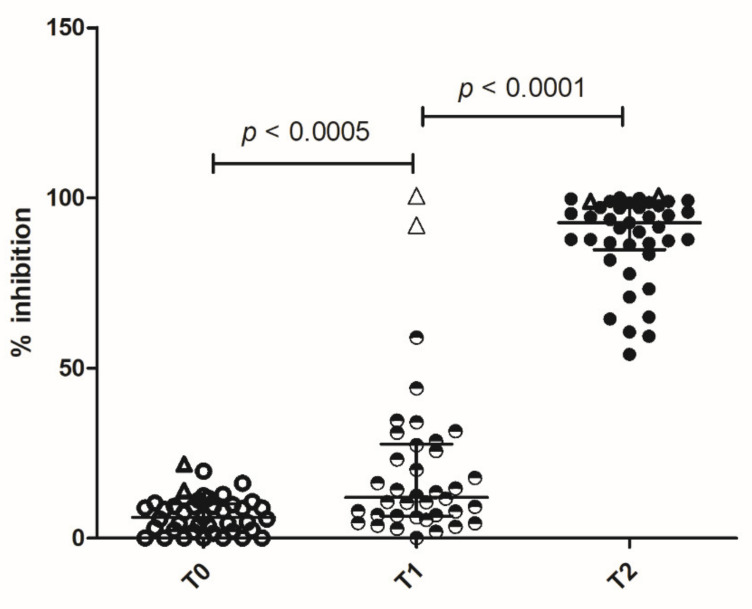
Spike protein inhibitory assay—neutralizing antibodies. Immunoglobulin neutralizing activity was measured by SPIA kit (DiaMetra Srl) at the baseline (T0), after the first dose of vaccine (T1) and after the booster (T2). Results are expressed as the percentage of inhibition of the binding of peroxidase-labeled ACE2 receptor to RBD-coated plates. After the second dose, all the subjects show higher neutralizing activity with respect to T1 and T0. Bars represent Median and interquartile range (IQR). The empty triangles represent the two vaccinated convalescent subjects. In these ones, high neutralizing ability is already achieved after the first dose.

A few subjects already produced antibodies after the first dose, inhibiting RBD interaction with ACE2. After the booster, high levels of inhibitory antibodies were detected in all the subjects (Figure 2). The two previously infected subjects had very high levels of inhibitory antibodies after the first dose of vaccine, which persisted after the booster (Figure 2).

### 3.3. Evaluation of Antibody Avidity Elicited by mRNA Vaccine

**Figure 3:**
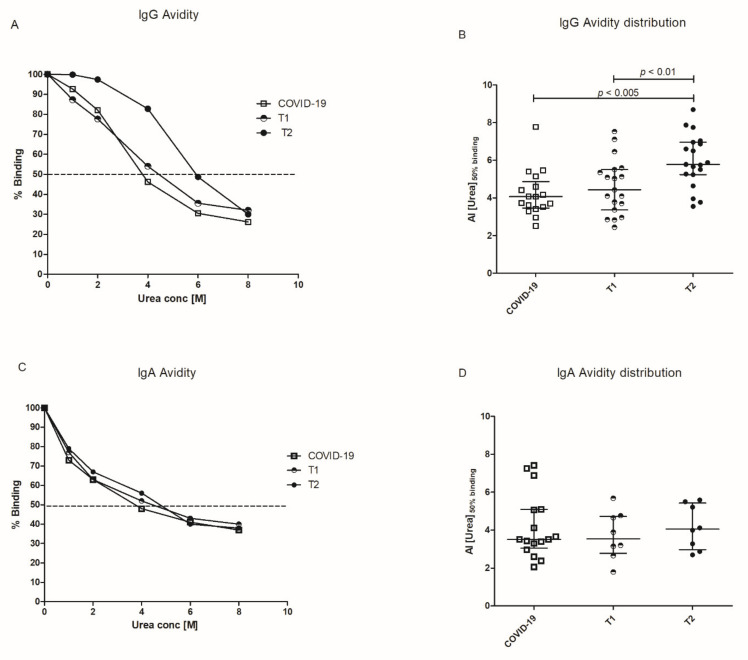
Avidity of anti-RBD IgG and anti-RBD IgA. Anti-RBD IgG (**A**,**B**) and IgA (**C**,**D**) avidity was measured by chaotropic ELISA using different urea concentrations. (**A**,**C**) Representative curves of IgG or IgA binding to RBD obtained with sera from a COVID-19 patient and a vaccinated healthcare worker after first dose (T1) and after booster (T2). (**B**,**D**) Distribution of Avidity Index (AI) calculated as urea concentrations that displaces the 50% of IgG or IgA binding in vaccinated at T1 and T2 and for comparison in ICU COVID-19 patients. In (**A**,**C**), the dotted line represents 50% of binding. In (**B**,**D**), Bars represent Median and interquartile range (IQR).

Antibody avidity was evaluated in a subgroup of 19 subjects by means of an avidity ELISA, in which antibody binding to solid phase RBD was disrupted by increasing concentrations of urea. Sera obtained after the first and the second dose of vaccine were analyzed and compared to sera from COVID-19 patients: representative examples are given in Figure 3A. The Avidity Index was expressed as the extrapolated urea concentration that displaces 50% of serum binding with respect to the control wells and results were given as [Median (IQR)]. In vaccinated subjects, antibody avidity increases after the booster [AI_T2_ = 5.78 (5.24–6.96); AI_T1_ = 4.43 (3.36–5.51)]. The antibody avidity in COVID-19 patients is also reported [AI = 4.07 (3.46–4.86)] (Figure 3B).

Among the 19 subjects evaluated for IgG avidity, we selected eight subjects that produced high amounts of anti-RBD IgA. In these sera, IgA avidity was tested by the same approach previously described. At variance with the IgG response, IgA avidity was not significantly different between T1 and T2 [AI_T1_ = 3.54 (2.77–4.73); AI_T2_ = 4.05 (2.97–5.42)] and between vaccinated subjects and COVID-19 patients [AI = 3.51 (3.04–5.09)] (Figure 3C,D).

## 4. Discussion

The data we obtained confirm that the BNT162b2 vaccine is highly effective in inducing both IgG and IgA anti-RBD antibodies, which are produced after the first dose and further increase after the second one. The production of high-titer IgA is of particular interest, for their potential protective role at the mucosal level. In this study, only serum IgA was detected but multiple reports indicate that the amount of antigen-specific secretory IgA is correlated with serum levels in COVID-19 patients [14,15]. Anti-RBD IgA was also identified in the saliva and milk of vaccinated subjects, suggesting the induction of an efficient local immunity [16,17].

The amounts of IgG and IgA anti-RBD antibodies far exceed those observed in COVID-19 patients, suggesting a highly efficient antigen production and presentation in all the subjects [18]

However, the titer of anti-RBD antibodies does not allow firm conclusions to be drawn on their role in protection from viral infection. It is well established that the immune response to RBD is polyclonal and not every antibody to RBD is expected to play a protective role. Plaque reduction neutralization tests represent the golden standard for the detection of neutralizing antibodies. However, a positive correlation between neutralization assays and inhibition of RBD–ACE2 interaction has been obtained by many authors [19,20] and the antibody-mediated blockage of ACE2–spike interaction has been proposed as a SARS-CoV-2 surrogate virus neutralization test [21,22].

Thus, we measured the potentially protective antibodies of any subclass that inhibits the interaction of RBD with ACE2. The high level of inhibition we observed by means of the SPIA assay indicates that the vaccine elicits protective antibodies, already after the first dose but in higher amounts after the booster.

The goal of vaccination is the induction of an immune response endowed with high specificity and high affinity. Thus, we further tested the quality of induced anti-RBD antibodies analyzing antibody avidity.

Avidity ELISA is a technique employed in a wide number of situations for the analysis of antibody avidity: during infections [23], in autoimmunity [24] and after vaccination [25]. Its correlation with more sophisticated and technically advanced methods for antibody analysis such as biospecific interaction analysis by surface plasmon resonance has been well documented [26].

As expected, affinity maturation takes place with boosting and the avidity assay we employed indicates a clear increase in IgG avidity with the number of immunizations. On the contrary, the increase in IgA avidity is less pronounced.

Overall, these results suggest that the two-dose strategy is able to achieve high levels of protective antibodies of high avidity in vaccinated subjects.

The main limitations of this study are represented by the number of subjects included and the short observation time, which does not permit any inference on vaccine efficacy over time. Antibody persistence will be analyzed in ongoing longitudinal studies, which allows the evaluation of the factors affecting magnitude and persistence of the immune response such as previous exposure to SARS-CoV-2 virus [27,28], age and gender [29,30].

A further limitation is the exclusive focus on humoral immunity, evaluated at the level of antibodies, without analyzing the B cell compartment functionally and phenotypically.

Further studies are needed to verify whether patients affected by autoimmune disorders or cancer patients display a similar response to mRNA vaccines and whether immunosuppressive treatment affects the qualitative or quantitative features of the immune response to vaccine.

## Data Availability

All data are available in the main text.

## Supplementary Materials

The following are available online at https://www.mdpi.com/article/10.3390/vaccines9060672/s1, Figure S1: Correlation between Anti Spike/RBD IgG Euroimmune and anti-RBD IgG home-made. Figure S2: End point dilution assays of Anti-RBD IgG, IgM, and IgA.
