# Supplementary material for: BNT162b2 mRNA SARS-CoV-2 Vaccine Elicits High Avidity and Neutralizing Antibodies in Healthcare Workers
Source: Vaccines (Basel). 2021 Jun 18;9(6):672. doi: 10.3390/vaccines9060672 (PMC8234791; doi:10.3390/vaccines9060672)
Supplement: Supplementary file 1 [file vaccines-09-00672-s001.zip › vaccines-1246032-supplementary.pdf]

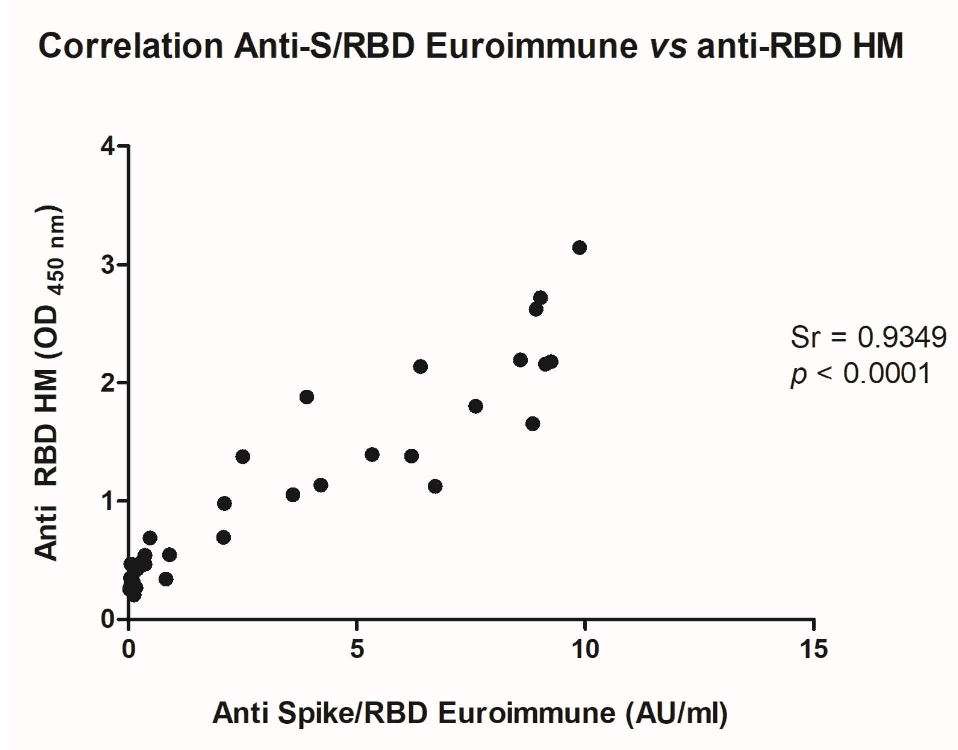

Figure S1. Correlation between Anti Spike/RBD IgG Euroimmune and anti-RBD IgG home made. The levels of anti-Spike/RBD IgG detected with a commercial assay and of anti-RBD IgG evaluated in house are reported respectively on x- and y- axis. Spearman rank correlation coefficient and *p*-value are reported.

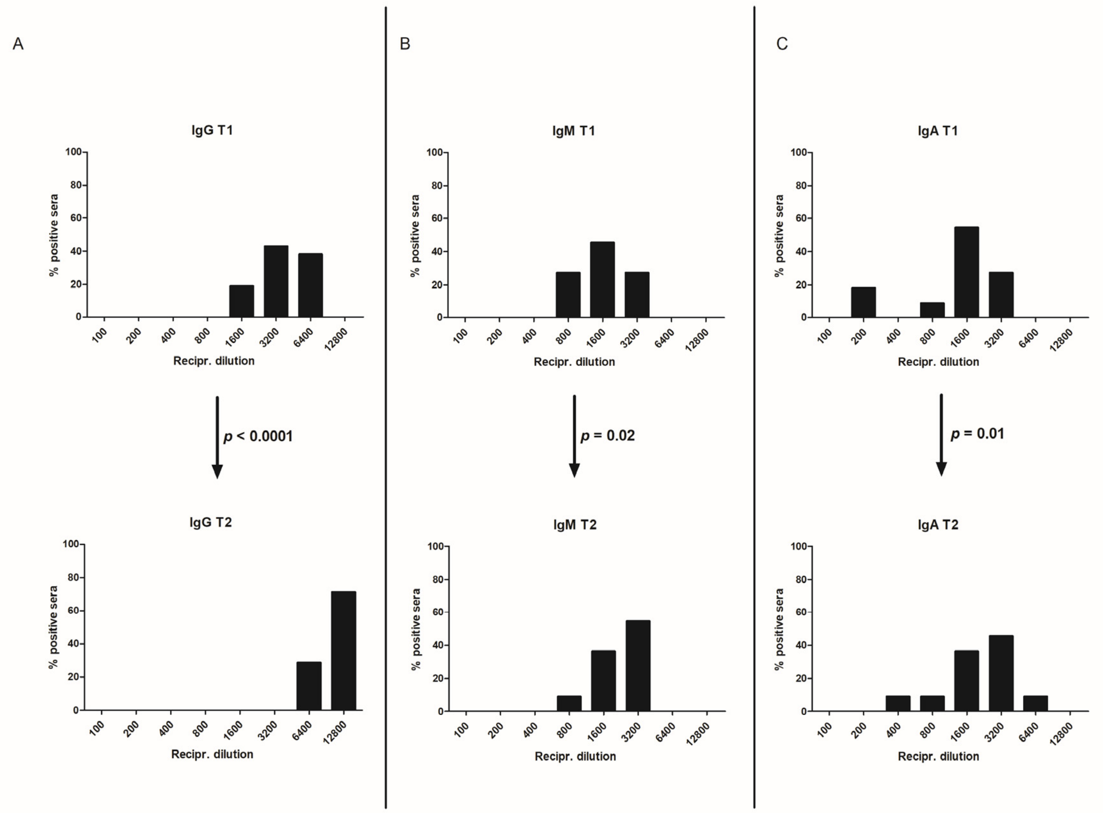

Figure S2. End point dilution assays of Anti-RBD IgG (**A**), IgM (**B**), and IgA (**C**). Antibody titers were also evaluated by means of endpoint dilution: IgG titers show a highly significative increase from T1 to T2 (**A**), whereas IgM and IgA undergo a minor (albeit present) increase (**B, C**).
